# Supplementary material for: SPECT/CT Imaging, Biodistribution and Radiation Dosimetry of a 177Lu-DOTA-Integrin αvβ6 Cystine Knot Peptide in a Pancreatic Cancer Xenograft Model
Source: Front Oncol. 2021 May 31;11:684713. doi: 10.3389/fonc.2021.684713 (PMC8200818; doi:10.3389/fonc.2021.684713)
Supplement: Supplementary file 1 [file DataSheet_1.docx]

**Supplementary Figures**

**Supplementary Figure S1**

**
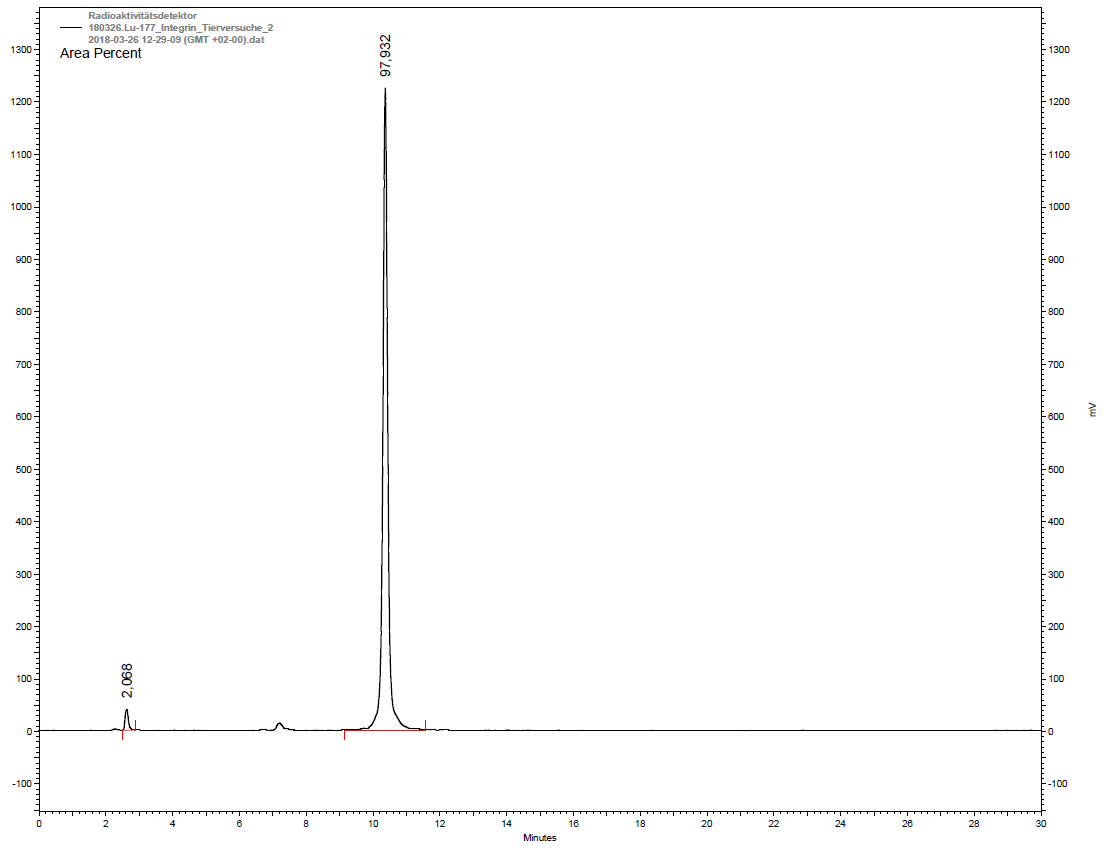
**

**Supplementary Figure S1: HPLC analysis.** HPLC analysis of ^177^Lu-DOTA-integrin αvβ6 knottin after radiolabeling demonstrated a purity of 97.9%.

**Supplementary Figure S2**

**
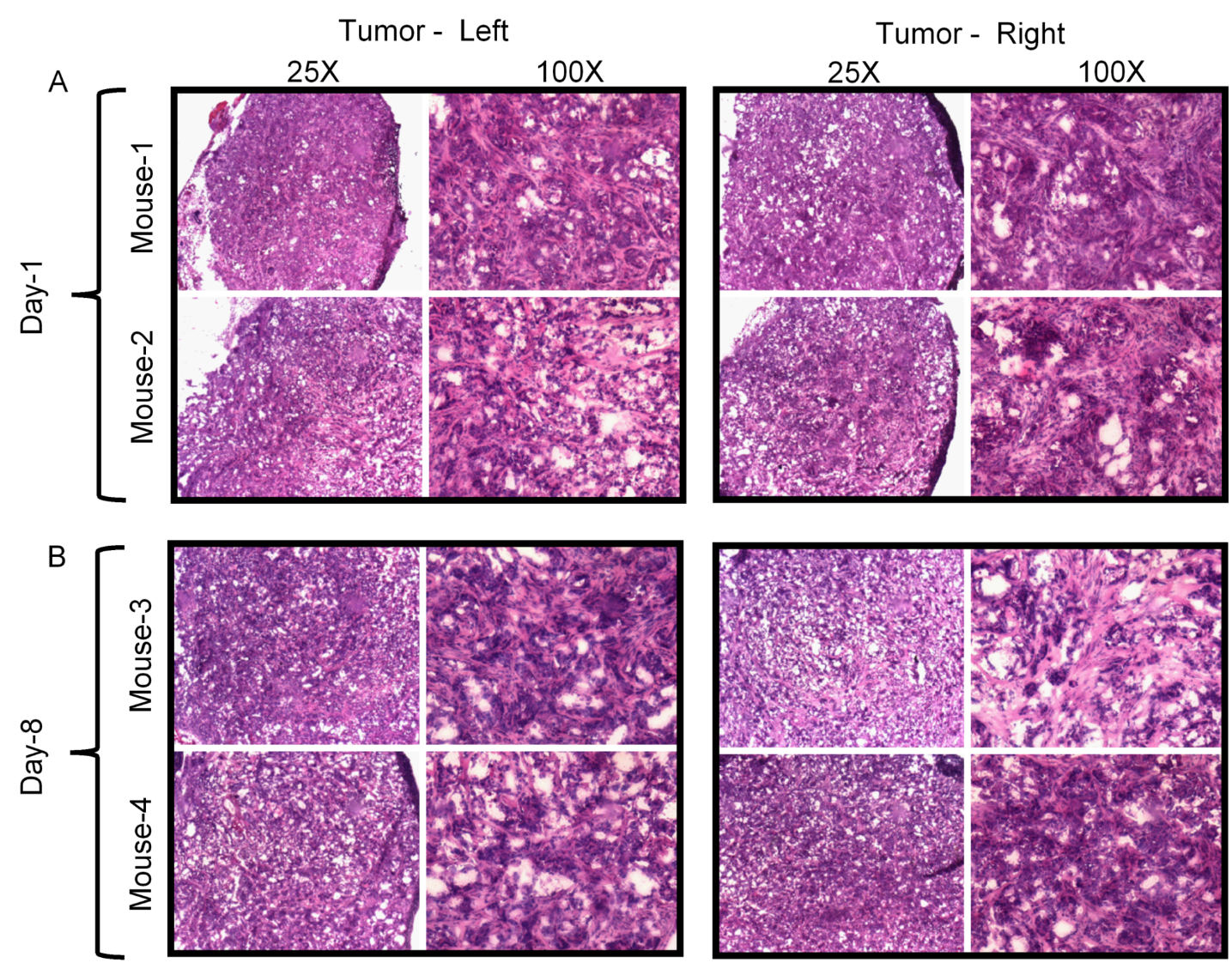
**

**Supplementary Figure S2: H&E staining of tumor tissue**. Comparison of H&E staining of the tumor (left and right) tissue from different mice at day-1 and day-8 post-injection
